# Use of LARS for soft tissue function reconstruction during tumor-type hemi-shoulder replacement achieves a good prognosis: a retrospective cohort study

**DOI:** 10.1186/s12957-023-03008-7

**Published:** 2023-04-04

**Authors:** Xiaopeng Tong, Hongbo He, Can Zhang, Yupeng Liu, Hao Zeng, Xinzhu Qiu, Qing Liu

**Affiliations:** 1 Department of Orthopaedics, Xiangya Hospital, Central South University, 87Th Xiangya Road, Changsha, 410008 Hunan China; 2 National Clinical Research Center for Geriatric Disorders, Xiangya Hospital, Changsha, Hunan 410008 People’s Republic of China

**Keywords:** LARS, Tumor-type hemi-shoulder replacement, Function reconstruction, Limb salvage, Muscle dynamic system

## Abstract

**Background:**

Shoulder soft tissue function reconstruction during tumor-type hemishoulder replacement is an important step to restore shoulder function. This study evaluates the functional prognosis and postoperative complications of ligament advanced reinforcement system (LARS)-assisted soft tissue functional reconstruction in tumor-type hemi-shoulder replacement.

**Materials and methods:**

Twenty-two patients with an average age of 37.5 ± 17.8 years diagnosed with benign invasive tumors, primary malignant bone tumors, or bone metastases were enrolled in this study. The patient’s medical records (history and surgical details), histological sections, imaging files, oncological prognosis, functional prognosis, and postoperative complications were collected. The upper limb function and shoulder joint function were evaluated using the Musculoskeletal Tumor Society (MSTS) system and American Shoulder and Elbow Surgeons (ASES) scoring criteria, respectively.

**Results:**

Twenty-two patients comprising 12 males and 10 females were enrolled. Overall, 9 patients had preoperative pathological fractures. The mean lesion length was 8.6 ± 3.0 cm. The local recurrence was observed in 3 cases, including 2 cases of osteosarcoma and 1 case of MGCT. A further 4 cases had pulmonary metastasis, including 2 cases with local tumor recurrence. The average postoperative MSTS score was 25.8 ± 1.7, and the score of postoperative ASES was 85.7 ± 6.0, both of which showed satisfactory functional recovery. Two cases experienced postoperative complications requiring surgical intervention, including one periprosthetic fracture and one giant cell granuloma. Prosthesis dislocation occurred in 1 case. None of the cases of periprosthetic infection or postoperative complications resulted in implant failure.

**Conclusions:**

LARS-assisted soft tissue function reconstruction in benign and malignant proximal humerus tumors after a tumor-type hemi-shoulder replacement is an effective technical improvement, which can effectively repair the integrity of the joint capsule to restore joint stability, provide a medium for soft tissue attachment to rebuild the muscular dynamic system, and eliminate residual dead space around the prosthesis, effectively improving limb function and reduce postoperative infection complications.

**Supplementary Information:**

The online version contains supplementary material available at 10.1186/s12957-023-03008-7.

## Introduction

The proximal humerus is a common site of primary malignant bone tumors and bone metastases, and surgical treatment of malignant tumors in this site has long been a difficult problem in orthopedics [1–3]. Further development and innovation in the fields of radiotherapy, chemotherapy, diagnostic imaging, and surgical techniques have provided new opportunities for limb salvage therapy for proximal humerus malignancies [4–7], allowing patients to retain greater limb function and improve the overall quality of life.

Limb salvage treatment of the proximal humerus is a major surgical challenge, as it involves both the repair of bone defects and the reconstruction of shoulder joint function [4, 8, 9]. The shoulder joint is the joint with the largest range of motion in the human skeleton, and the rotator cuff and joint capsule are critical to its stability. As proximal humerus tumors mostly involve the surrounding soft tissue structure including the rotator cuff, leading to the sacrifice of the functional soft tissue stability structure around the glenohumerus joint, reconstruction of shoulder joint function is as important as the repair of bone defects in limb salvage treatment [10, 11].

The most commonly used techniques for bone defect repair after resection of proximal humerus tumors include osteoarticular allografts [11, 12], allograft-prosthetic composites (APC) [13, 14], and modular prosthesis [15, 16]. Allogeneic arthrodesis, vascularized fibular transfer, and scapular replacement are also occasionally used as alternatives [2, 17]. Although osteoarticular allograft and APC can provide a healing interface for the reconstruction of the dynamic stable structure of the shoulder joint, postoperative complications caused by transplantation itself, such as graft fracture, bone nonunion, rejection, cartilage surface collapse, and other problems, lead to a high reoperation rate and a gradually decreasing graft survival rate [11–14, 18], which gradually weakens the clinical application of this technique. Coincidentally, because of its flexibility, relatively low complication rate, high implant survival rate, and acceptable and repeatable functional results, modular prosthesis reconstruction has gradually come to occupy the main position in clinical application [19, 20]. However, since the shoulder capsule and rotator cuff cannot be firmly fixed directly on the prosthesis surface to promote biological healing, joint function reconstruction after modular prosthesis replacement is also a significant clinical challenge.

At present, there are no normative guidelines for functional reconstruction after tumor-type hemi-shoulder replacement in clinical practice. In the early stage of the clinical application of biological mesh, researchers mostly used simple mechanical sutures to reconstruct shoulder joint capsules and tendon insertions [9, 10]. With the advent of various biologic mesh implants, researchers have found that patients who received biologic mesh for soft tissue reconstruction have better shoulder joint function than those who did not receive synthetic mesh [13, 15, 21]. With the evaluation of the biocompatibility of biological mesh and the effect of soft tissue implantation, people’s requirements for biological materials are further improved, and various bone tumor centers continue to innovate and improve themselves on the optimization of shoulder joint functional reconstruction according to previous experience.

Ligament advanced reinforcement system (LARS) is a new biomaterial with good histocompatibility, low aseptic inflammatory response, anti-infectivity, and good stiffness, tension, and elasticity [22]. Its advantages in cruciate ligament reconstruction and tendon reconstruction have been widely known. Our bone tumor center has also been using LARS for ligament reconstruction, joint capsule repair, and soft tissue reconstruction after prosthesis replacement.

We speculate whether LARS has an advantage over other biological patches in the reconstruction of soft tissue function after half-shoulder joint replacement. Therefore, in the present study, we investigated the clinical effect of LARS-assisted joint functional reconstruction in tumor-type hemi-shoulder replacement in our bone tumor center. The objective is to evaluate the true clinical effect of LARS-assisted joint functional reconstruction from the degree of postoperative joint functional recovery and the incidence of postoperative complications. The survival rate of the prosthesis, the degree of recovery of joint function, and the incidence of complications were followed up and analyzed.

## Materials and methods

### Patients

We retrospectively evaluated patients who underwent segmental resection of proximal humeral tumors and modular tumor-type hemiarthroplasty at our bone tumor center from January 2012 to January 2022. All patients were treated according to the preoperative consultation opinions from our bone tumor center’s multi-disciplinary team (MDT).

The inclusion criteria were as follows: (1) patients who underwent proximal humerus hemiarticulectomy, (2) modular hemiarticular prosthesis was used for reconstruction, (3) LARS-assisted joint functional reconstruction was performed, (4) the axillary nerve and most deltoid muscles were preserved, (5) complete clinical data was available, and (6) postoperative follow-up was > 12 months. The exclusion criteria were (1) underwent total humerus resection, (2) underwent extra-articular resection, or (3) underwent revision surgery.

This study was conducted according to the Declaration of Helsinki and approved by the clinical medical research ethics committee of Xiangya Hospital of Central South University. All patients participating in the study or their legal guardians received and signed informed consent.

**Fig. 1:**
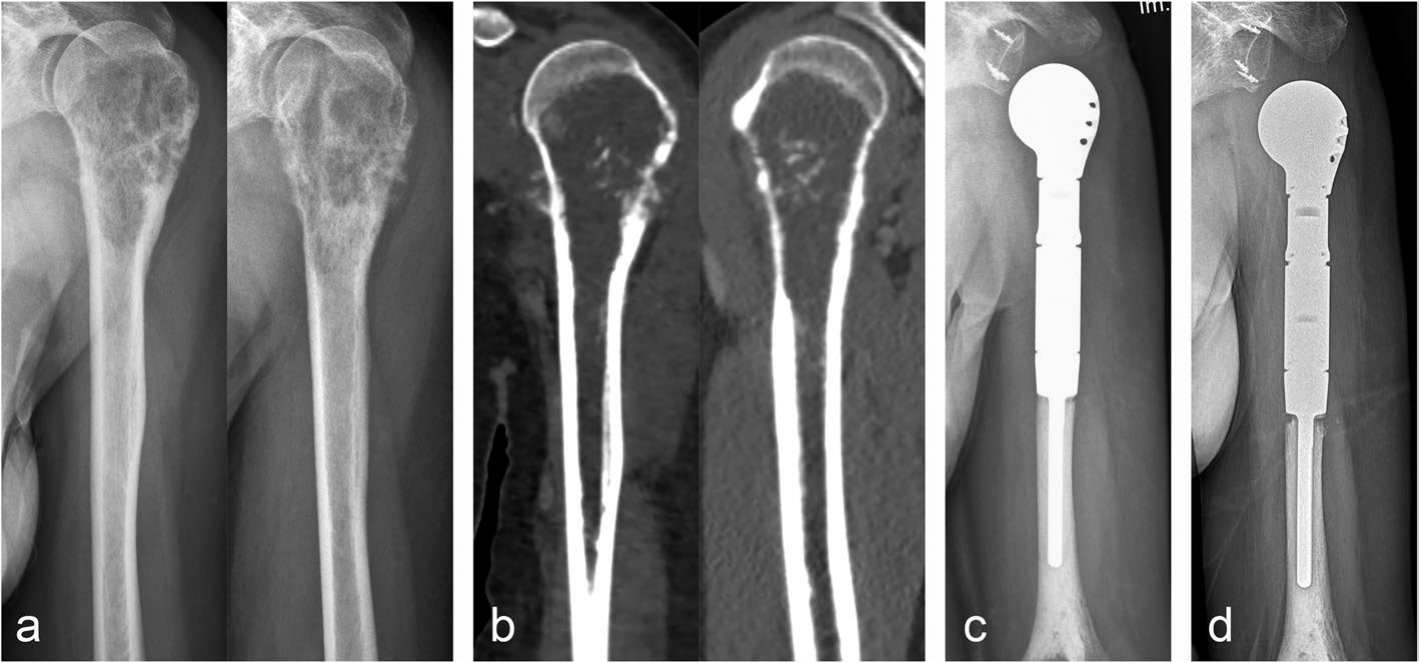
En bloc resection of osteosarcoma and reconstruction of tumor-type hemi-shoulder replacement. **a** Preoperative anteroposterior and lateral X-rays. **b** Coronal and sagittal CT scans showed extensive bone destruction with periosteal reaction. **c** Anteroposterior X-ray three months after surgery showed that the prosthesis was firmly fixed and the joint was stable. **d** The slightly downward displacement of the prosthesis was found at 1 year postoperatively

**Fig. 2:**
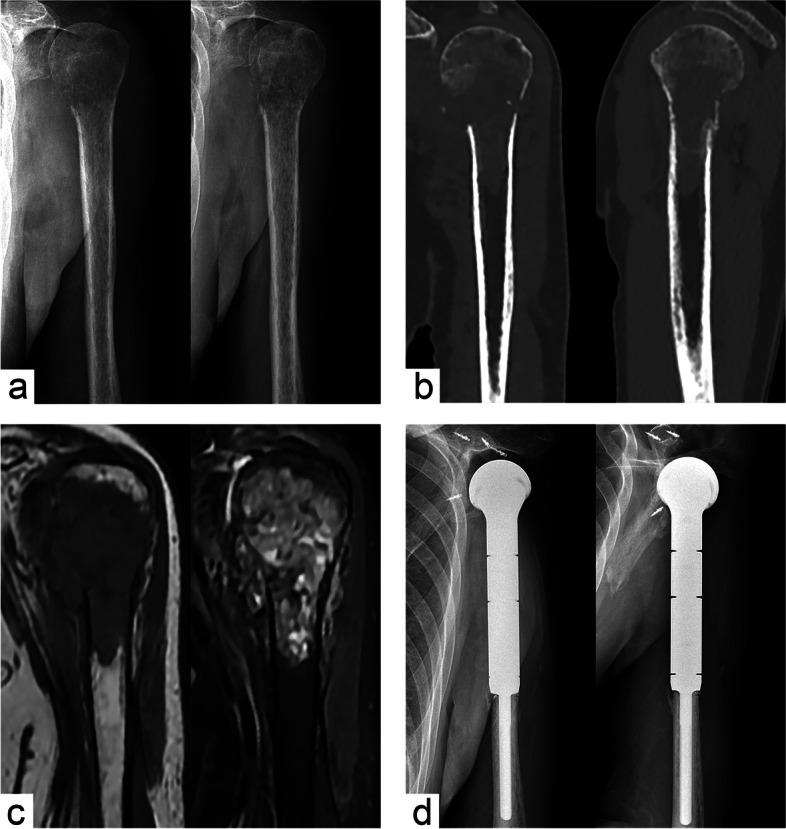
Segmental resection and hemi-shoulder replacement for bone metastases. **a** Preoperative anteroposterior and lateral X-rays indicated an osteolytic lesion. **b** CT scan indicated extensive bone erosion and pathological fracture. **c** MRI suggested that the lesion involved surrounding soft tissues. **d** Postoperative anteroposterior and lateral X-rays showed that the prosthesis was securely fixed and the joint was stable

**Fig. 3:**
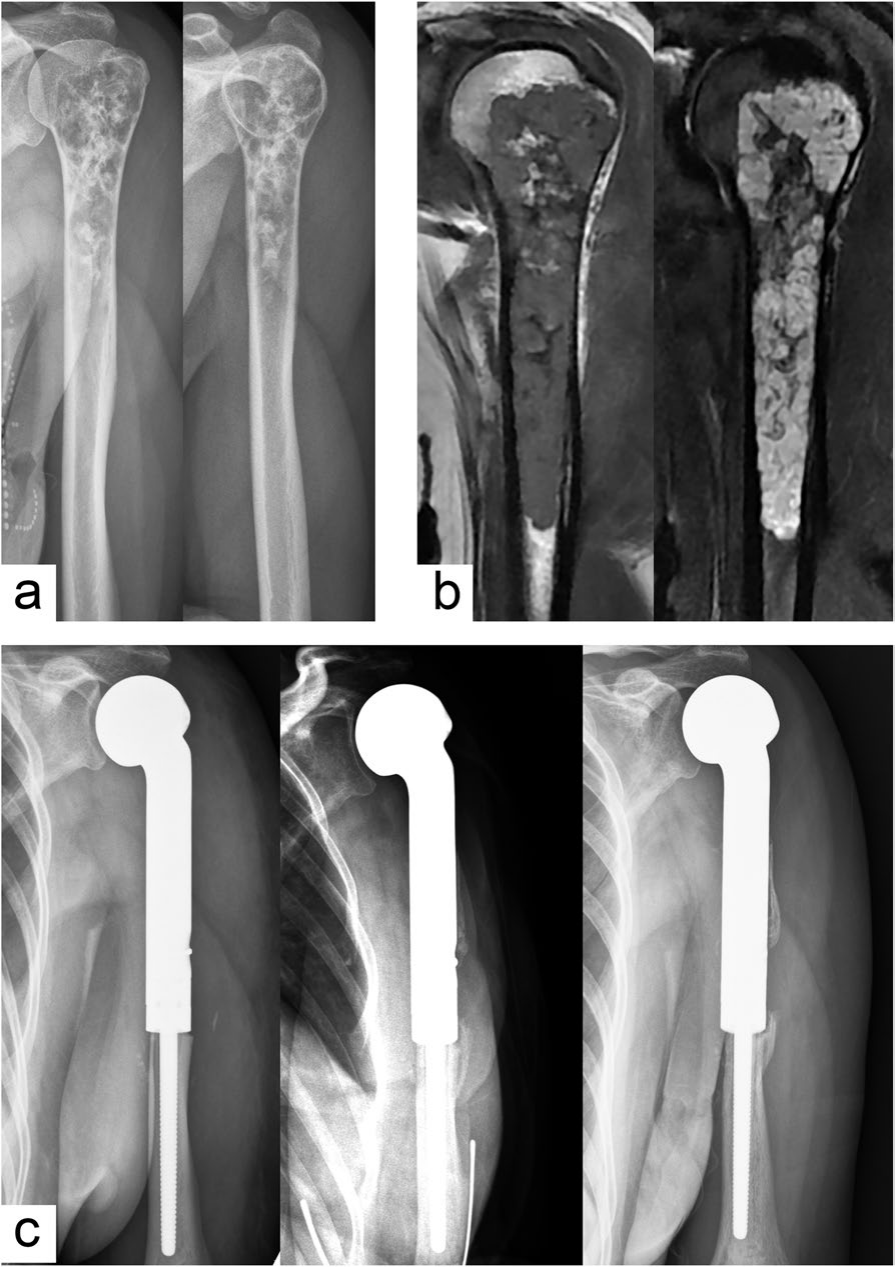
Segmental resection and hemi-shoulder replacement for high-grade chondrosarcoma. **a** Preoperative anteroposterior and lateral X-rays. **b** MRI showed extensive cortical involvement and heterogeneous signals within the tumor. **c** Postoperative anteroposterior X-rays of 1 week, 1 year, and 3 years showed that the prosthesis was in a stable position

A total of 22 patients who met the criteria with an average age of 37.5 ± 17.8 years were enrolled in our cohort. Pathologic diagnoses included giant cell tumors of the bone (*n* = 6), osteosarcoma (*n* = 5) (Fig. 1), bone metastases (*n* = 4) (Fig. 2), chondrosarcoma (*n* = 3) (Fig. 3), lymphoma (*n* = 1), Ewing’s sarcoma (*n* = 1), chondroblastoma (*n* = 1), and malignant giant cell tumors of bone (MGCT) (*n* = 1). According to the property and characteristics of the tumor, all patients with osteosarcoma and Ewing’s sarcoma received standardized preoperative and postoperative chemotherapy. One patient with lymphoma was transferred to the hematology department for standardized postoperative chemotherapy. None of the other patients received chemotherapy or radiation. Four patients with bone metastases received postoperative targeted drug therapy. A follow-up was performed, and the patient’s medical records (medical history, imaging files, and surgical details) (Table 1), tumor prognosis, functional prognosis, and postoperative complications were collected.

**Table 1:** Demographic variables

No	Gender	Age (years)	Pathological classification	Side	Tumor length (cm)	Pathological fracture (Y or N)	Enneking staging	Body mass index
1	M	25	MGCT	Right	11	Y	IIIB	25.3
2	M	55	Bone metastase	Left	9	Y	IIIB	18.9
3	F	42	GCT	Left	5.5	Y	stage 3	21.5
4	F	15	GCT	Right	5.5	N	stage 3	23.3
5	F	38	GCT	Right	8	N	stage 3	20.8
6	M	24	GCT	Right	8	Y	stage 3	19.5
7	M	47	GCT	Left	8.5	Y	stage 3	25.4
8	M	25	GCT	Left	7.5	Y	stage 3	25.1
9	F	13	Osteosarcoma	Right	12	N	IIB	17.8
10	F	32	Osteosarcoma	Right	11	N	IIIB	23.5
11	F	48	Osteosarcoma	Right	6.5	N	IIB	20.9
12	M	14	Osteosarcoma	Left	15	N	IIB	21.6
13	M	18	Osteosarcoma	Left	10	N	IIB	23.3
14	F	78	lymphoma	Left	13	Y	IIIB	18.5
15	F	48	Bone metastase	Right	5	N	IIIB	20.3
16	M	13	Chondroblastoma	Right	3.5	N	stage 3	22.4
17	M	47	Chondrosarcoma	Right	10.5	N	IB	19.8
18	M	45	Chondrosarcoma	Right	8	N	IIB	21.3
19	M	31	Chondrosarcoma	Right	7	N	IIB	23.5
20	F	51	Ewing’s sarcoma	Left	13	N	IIB	20.7
21	F	56	Bone metastase	Left	7	Y	IIIB	23.3
22	M	60	Bone metastase	Left	5.5	Y	IIIB	18.8

*MGCT* malignant giant cell tumors of the bone, *GCT* giant cell tumors of the bone

### Operative technique

The tumor resection boundary and osteotomy length were determined by preoperative enhanced MRI. All operations involving tumor resection and reconstruction were performed collaboratively by our treatment group. All patients underwent intraarticular tumor resection of the proximal humerus.

**Fig. 4:**
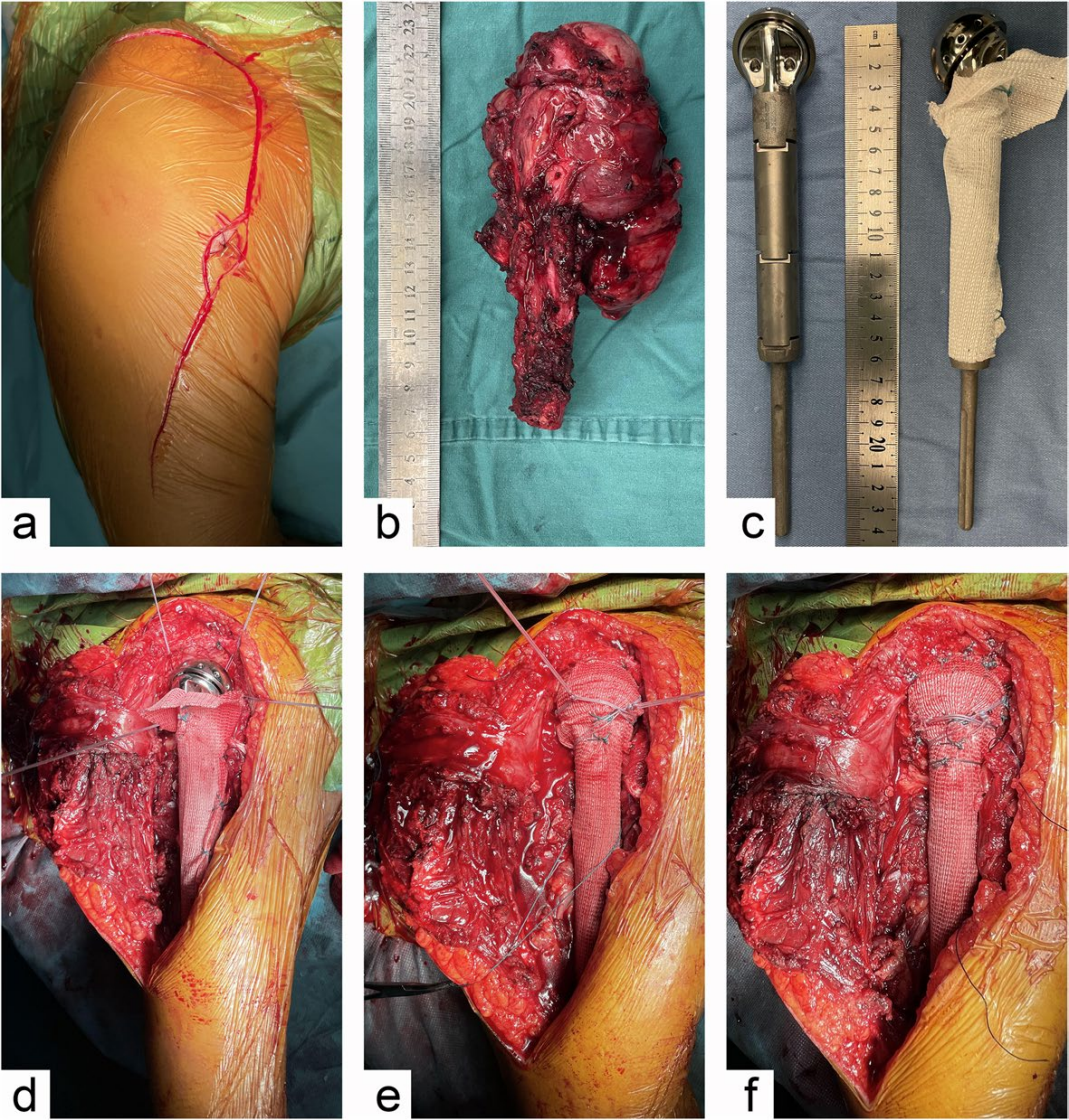
Operative details of tumor-type hemi-shoulder replacement in proximal humerus (**a**) using Henrey incision and removing biopsy trajectory. **b** General picture of the removed tumor segment. **c** Modular tumor prosthesis and LARS. **d** Place four rivets into the scapular glenoid from different directions. **e** Stitch the rivet thread securely to LARS. **f** The stump of the joint capsule was sutured to LARS

Tumors were removed using the Henrey incision, and the trajectory of the biopsy was removed (Fig. 4a). The upper arm medial deltoide-pectoralis major intersulci were followed and cut outward from the deltoide-origin at the coracoid process while paying attention to protecting the cephalic veins. The proximal humerus was exposed to the shoulder joint and the insertion of the pectoralis major, latissimus dorsi, and teres major was severed. The rotator cuff muscles around the shoulder joint were exposed and severed successively, while the long head tendon of the biceps was severed, and the shoulder capsule was severed on the side of the glenoid. The residual posterior muscle attachment was dissociated, with attention paid to the correct dissociation and protection of the radial neurovascular bundle, while the proximal humerus was completely dissociated. Osteotomy was performed on the distal end of the humerus at an extension of 3–5 cm according to the preoperative design (Fig. 4b).

After the length of the prosthesis was determined by the reaming mold, the prosthesis (THYTEC, Shanghai, China) was fixed in the distal medullary cavity with bone cement. LARS wrapping prosthesis was used and properly fixed (Fig. 4c). For patients with large soft tissue defects, a rivet was used to assist in the repair of the shoulder joint capsule. Four rivets were inserted into the glenoid fossa from different directions (Fig. 4d) and securely sutured with LARS (Fig. 4e), and then, the residual end of the shoulder joint capsule was closely sutured with LARS to repair the integrity of the joint capsule (Fig. 4f). With LARS as the carrier, the rotator cuff, deltoid insertion, long head tendon of biceps brachii, pectoralis major, latissimus dorsi, and other muscle stumps were sutured on LARS according to their anatomical positions. Two wound drainage tubes were retained, the deep fascia was continuously sutured, and finally, the incision was closed layer by layer.

### Postoperative management and follow-up

All patients underwent standardized postoperative rehabilitation procedures. A shoulder abduction fixator was worn for 4 weeks while wrist and forearm contractions were performed. Subsequently, the physiotherapist instructed the patients to initiate passive functional exercise, focusing on the forward bending and abduction of the shoulder. Patients were followed up at 6 and 12 weeks postoperatively, and then, every 3 months for the first 2 years, every 6 months for the next 3 years, and annually thereafter. During follow-up, clinicians mainly evaluated the oncological prognosis and functional prognosis and recorded the occurrence of various complications. Postoperative limb function was assessed using the Musculoskeletal Tumor Society (MSTS) system [23]. Meanwhile, assessment of activities of daily living (ADL) involving the shoulder using American Shoulder and Elbow Surgeons (ASES) scoring criteria [24]. The pain was graded using the visual analog scale (VAS) [25].

### Statistical analysis

Data analyses were performed using SPSS version 26.0 (SPSS Inc., Chicago, Illinois). Measurements were expressed as mean ± standard deviation. Follow-up data were analyzed by paired or unpaired *t* test. A *P* value < 0.05 was considered statistically significant.

## Results

For this study, we enrolled 22 patients, including 12 males and 10 females, with an average age of 37.5 ± 17.8 years. The lesion was located on the right side in 12 patients and on the left side in 10 patients. The mean body mass index was 21.6 ± 2.3. Nine patients had preoperative pathological fractures (Fig. 2). The mean lesion length was 8.6 ± 3.0 cm. The Enneking stage [26, 27] of tumors in this cohort was grade 3 (7 cases), IIB (8 cases), and IIIB (7 cases), and the mean postoperative follow-up was 48.2 ± 27.8 months (Table 1).

Of the 22 patients in this study, 6 died at the last follow-up, including 2 patients with osteosarcoma and 4 patients with bone metastases. The local recurrence was observed in 3 cases, including 2 cases of osteosarcoma and 1 case of MGCT. These patients eventually underwent joint amputation. Among all cases with primary tumors, 4 cases had pulmonary metastasis during follow-up, including two cases with local recurrence who developed multiple lung metastases after surgery. Two other cases with single lung metastasis survived after resection of the metastatic lesion. None of the four patients with bone metastasis in this cohort underwent reoperation at the original surgical site at the final follow-up, and the follow-up time was more than 12 months in all cases.

**Fig. 5:**
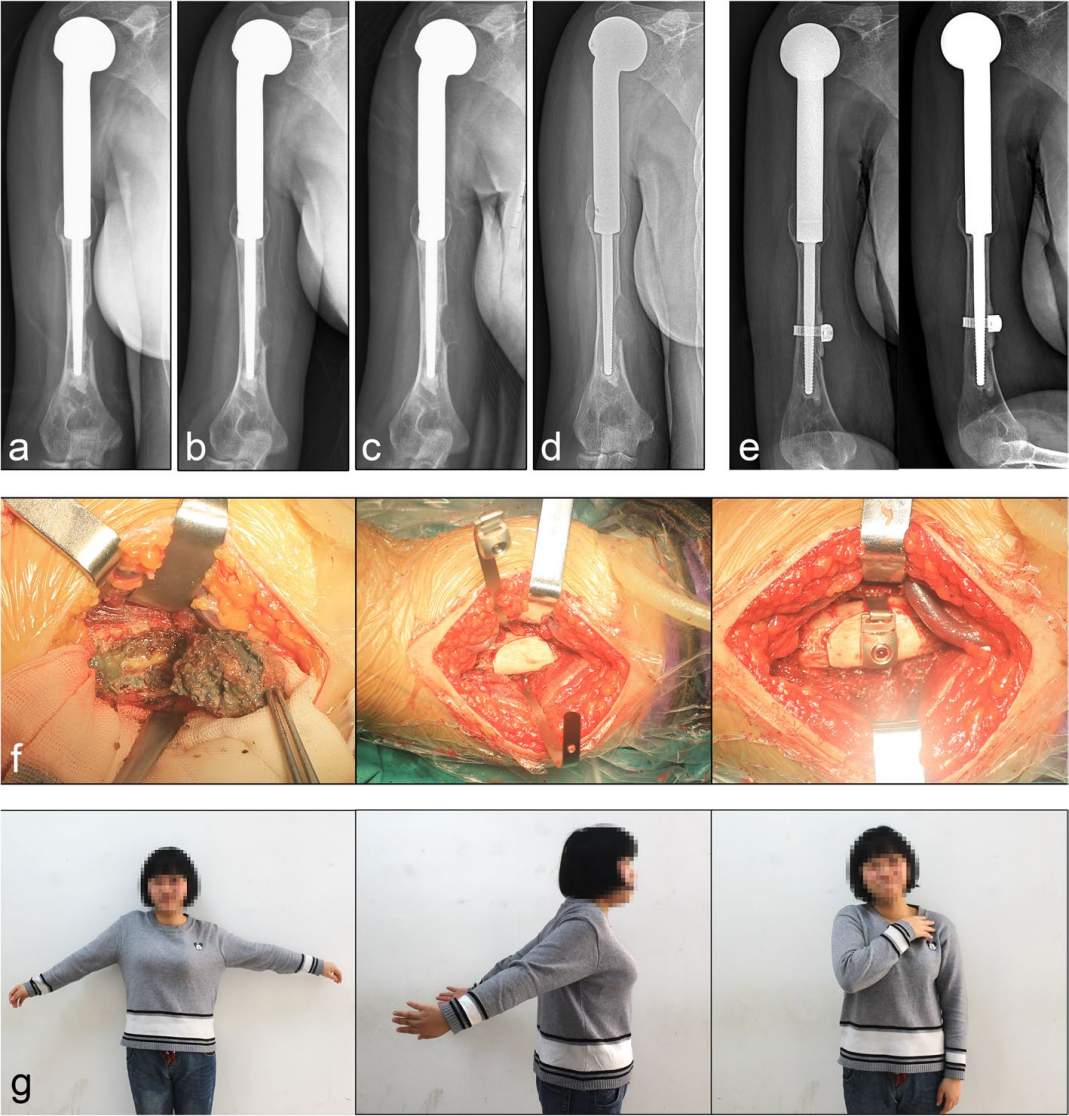
Giant cell granuloma of distal prosthesis was observed during follow-up. **a**–**d** Four consecutive X-ray reexaminations revealed osteolytic lesions of the distal humerus with slight enlargement. **e** Postoperative X-rays of lesion resection and fixation of allograft bone block with bone cerclage. **f** The lesion was excised, then an allograft of appropriate size was cut to repair the bone defect, and finally, the allograft was fixed with a bone cerclage. **g** Postoperative shoulder joint motion was satisfactory, especially shoulder abduction function

The postoperative MSTS score of patients in this cohort was 25.8 ± 1.7, showing a significant difference from that before surgery (*p* < 0.05). The postoperative ASES was 85.7 ± 6.0, of which 20 cases (90.9%) showed good recovery of shoulder joint movement, ability of ADL, and satisfactory recovery of upper limb muscle strength (Fig. 5). One case (4.5%) developed forward and upward prolapse of the prosthesis after the operation, which resulted in obvious limited shoulder joint activity, and no surgical treatment was performed. One case (4.5%) of osteosarcoma with the partial sacrifice of the musculocutaneous nerve and median nerve experienced poorer upper limb function and muscle strength than the healthy side after surgery. Postoperative pain scores in this cohort were significantly improved compared with those before surgery (*p* < 0.05) (Table 2).

**Table 2:** Statistics of clinical information and follow-up data

**General information**		**Mean**	**SD or percentage**
*Duration of follow-up (month)*		48.2	27.8
*Postop ASES score*		85.7	6.0
*ADL score*		33.2	1.6
*ROM (°)*	Flexion	75.4	15.9
	Abduction	55.9	13.9
	Extension	36.0	5.3
	Internal rotation	48.4	7.3
	External rotation	76.1	8.6
*Complications*	Recurrence	3	13.6%
	Dislocation	1	4.5%
	Periprosthetic fractures	1	4.5%
	Giant cell granuloma	1	4.5%
**Comparative analysis**	**state**	**Mean ± SD**	***P* value**
*MSTS score*	Preoperative	15.4 ± 6.1	< 0.001
	Postoperative	25.8 ± 1.7	
*VAS score*	Preoperative	4.2 ± 1.6	< 0.001
	Postoperative	0.3 ± 0.6	

*ASES *American Shoulder and Elbow Surgeons, *ADL* activities of daily living, *ROM *range of motion, *MSTS* Musculoskeletal Tumour Society Scoring System, *VAS* visual analog scale

Two cases in this cohort had postoperative complications requiring surgical intervention, including one periprosthetic fracture and one giant cell granuloma (Fig. 5). After surgical treatment, the prosthesis was retained and the function of the shoulder joint recovered satisfactorily. In addition, none of the cases in this cohort had a periprosthetic infection or postoperative complications leading to implant failure.

## Discussion

In this study, we reviewed the clinical data of patients with joint function reconstruction using LARS-assisted tumor-type hemi-shoulder replacement in our center in the past 10 years. In our cohort, the survival rate of prostheses after surgery was 100%, only 1 case of shoulder dislocation occurred, and the postoperative MSTS score was 25.8 ± 1.7. Overall, 90.9% of patients were able to return to normal daily activities and upper limb muscle strength. Despite the occurrence of two complications requiring surgical intervention, both implants were preserved and functional recovery was satisfactory after active management. In general, the results of this study are superior to previous studies in terms of shoulder dislocation rate, shoulder joint range of motion, postoperative infection, and other complications. It is not difficult to show that LARS plays an important role in the reconstruction of shoulder joint function.

The choice of reconstruction method for proximal humerus limb salvage therapy depends on the properties and size of the tumor, the degree of excision required to obtain a wide and clear incisal margin, the resources available, and the surgeon’s choice and familiarity with the potential procedures [2, 11]. Due to its accessibility, low complication rate, and high graft survival rate, modular prosthesis reconstruction has gradually replaced osteoarticular allograft and APC reconstruction as the most mainstream bone defect repair option [15, 18]. However, the joint capsule and rotator cuff cannot be attached to the surface of the prosthesis, which leads to a decrease in the range of motion and stability of the joint, posing a new challenge for joint function reconstruction.

Because the diameter of the humeral head is larger than that of the glenoid, the shoulder joint is both the most mobile and unstable joint in the human body [28]. Its stability depends on the deepening of the glenoid lip to form the ball-socket joint and the rotator cuff formed by the soft tissue around the joint. During proximal humeral prosthesis replacement, the prosthetic humeral head and scapular glenoid are not absolutely matched, and it is difficult to achieve contact stability. Furthermore, there is no biological healing between the soft tissue and the prosthesis [15, 21], and the structure is not as strong as the tendon-bone connection. Therefore, reasonable repair of shoulder joint static stability structure and reconstruction of the muscle dynamic system is very important to restore shoulder joint function.

The core of functional reconstruction of the shoulder joint lies in the biological attachment of soft tissue on the surface of the prosthesis. Therefore, researchers have made many attempts to functional reconstruction in previous studies. Degeorge et al. [13] used APC transplantation to achieve optimal humerus height recovery and rotator cuff tendon reinsertion and found high postoperative complications and unsatisfactory recovery of joint function. Marulanda et al. [21] used aortograft mesh to reconstruct shoulder joint function, and the incidence of postoperative complications was significantly reduced, but the abductor function of the shoulder joint was not satisfactory. Wang et al. [15] used polypropylene mesh-wrapped prosthesis to assist soft tissue function reconstruction, which also effectively controlled the occurrence of postoperative complications, but the recovery of joint function was still not satisfactory. These improvements effectively promote the innovation of functional reconstruction of soft tissue and also made the surgeon realize the importance of the strength and biocompatibility of the biological patch for functional reconstruction.

LARS is a porous material made of polyethylene terephthalate, with a minimum failure load of 4000N, which has excellent biocompatibility, traction resistance, corrosion resistance, and the ability to stimulate the rapid growth of fibroblasts [22, 29, 30]. Its porous design allows for the multi-point fixation of muscle attachments, which not only distributes stress and reduces tearing, but also promises to improve the current situation where tumor-type prosthesis can only provide point-like or linear muscle attachment points, restoring the anatomical fixation of their planar attachment to the humerus [22].

The stability of the joint structure is the premise of restoring limb function, and the integrity of the joint capsule structure can effectively prevent joint dislocation after the operation [20, 31]. It is important to consider that tumor resection of the proximal humerus is often accompanied by the loss of all or part of the joint capsule and surrounding muscle tissue, which forms a huge soft tissue defect and destroys the static stability of the joint structure and muscle attachment site. As such, many attempts have been made to reconstruct the stability of the shoulder joint. Mayilvahanan et al. [32] used stainless steel wire or dacron tapes for the static suspension to fix the proximal acromion process of the prosthesis, and postoperative joint dislocation occurred in 6 cases, achieving a postoperative functional satisfaction rate of 78%. Cannon et al. [10] used polyester tape or polyester braided sutures to suture the residual joint capsule to the sagittal hole of the prosthesis to stabilize the shoulder joint. Radiological evidence showed that 22 patients (29%) subsequently experienced proximal displacement of the prosthesis. These techniques all involved mechanical riveting fixation, which cannot truly achieve reattachment of soft tissue on the prosthesis. Therefore, using LARS as a carrier to assist the realization of biological attachment of the shoulder soft tissue in a prosthesis may indeed be an ideal treatment option to restore shoulder joint stability. In the technique described herein, the tubular structure of LARS is used to replace the joint capsule, and the periphery is sutured to the stump of the joint capsule or the glenoid fossa. Meanwhile, rivets can be implanted at different positions of the glenoid fossa to strengthen the repair of the joint capsule from different angles and to restrict the activities of the humeral head and effectively prevent dislocation of the shoulder.

The reconstruction of joint function is closely related to both the restoration of the integrity of the joint capsule structure, and the restoration of the muscle dynamic system, which can achieve dynamic stability of the joint through mechanical action. The most important of these is the rotator cuff tissue attached to the greater and lesser tubercles, followed by the deltoid and biceps longhead tendons which also play an important role in the stability of the joint. LARS provides a carrier for muscle tissue to attach to the surface of the prosthesis, and it can achieve rapid adhesion and healing with muscle tissue into a structural unit to restore the integrity of the muscle dynamic system and strengthen force transmission [16, 33]. LARS not only ensures the soft tissue coverage of the prosthesis, but also prevents the power loss caused by extensive tumor resection to the greatest extent possible, achieving a biomechanical balance around the joint, realizing the recovery of limb function, and further enhancing the stability of the joint.

When we focus on the contribution of LARS to the recovery of joint structure and functional reconstruction, we often neglect its positive role in reducing the incidence of postoperative complications [29]. As an artificial material, LARS is a foreign body, the implantation of which theoretically increases the risk of deep infection of prosthesis. However, the results of this study showed the opposite. The application of LARS greatly reduced the incidence of postoperative periprosthesis infection, which may be inseparable from the fact that LARS could effectively increase the soft tissue coverage on the prosthesis surface and eliminate the dead space in the operative area. The presence of LARS can realize the attachment of muscle residues of the proximal humerus, including the biceps brachii, joint tendon, pectoralis major, subscapularis, and teres major on the surface of the prosthesis; increase the effective coverage of soft tissue on the surface of the prosthesis; eliminate the dead space caused by muscle retraction; avoid postoperative deep fluid accumulation in the operative area; and eliminate the risk factors of deep infection [33, 34].

Through this study, we found that LARS indeed provided effective help for the functional reconstruction of the proximal humerus tumor-type hemi-shoulder replacement. This can not only effectively repair the integrity of the shoulder capsule and rebuild the muscular dynamic system of the shoulder joint, but also effectively reduce the probability of postoperative deep infection and extend the survival time of the prosthesis. Considering the continuous progress of medicine, with the development of surgical technology, optimization of prosthesis design, and innovation of medical materials, patients will benefit more from limb salvage methods in the future. It is also hoped that our research can provide some experience and data support for limb salvage treatment of proximal humeral tumors.

Nevertheless, this study has some limitations which should be mentioned. As a single-center retrospective study, the small sample size and lack of effective controls are the fundamental defects in the study design. Furthermore, the follow-up period is insufficient to accurately evaluate the mechanical-related complications. In addition, due to the differences in tumor properties, there are individual differences in the scope of tumor resection, and the evaluation of postoperative functional recovery cannot be uniform. These shortcomings will be investigated in our future studies.

## Conclusions

In conclusion, LARS-assisted soft tissue function reconstruction in benign and malignant proximal humerus tumors after a tumor-type hemi-shoulder replacement is an effective technical improvement, which can repair or strengthen the static stable structure of the joint, rebuild the muscle dynamic system, and provide a new medium for soft tissue reattachment on the surface of the prosthesis. Furthermore, this technique induces a reasonable improvement in postoperative joint function and reduces the incidence of postoperative complications.

## Supplementary Information

**Additional file 1: Figure S1.** Postoperative follow-up revealed complications of prosthesis dislocation. (a) X-ray review indicated that the joint structure was stable at 6 months postoperatively. (b) Forward and upward dislocation of the prosthesis was found at 1 year postoperatively. (c) The red arrow indicates a dislocated humeral head in the patient's frontal view.

**Additional file 2: Table S1.** MGCT, malignant giant cell tumors of bone; GCT, giant cell tumors of bone.

## Data Availability

All the data used in the article can be obtained from the medical record information system of Xiangya Hospital, Central South University. Any questions or inquiries regarding the present study can be directed to Qing Liu, MD, PhD (158112225@csu.edu.cn), as the corresponding author.

## Abbreviations

LARS: Ligament advanced reinforcement system
APC: Allograft-prosthetic composites
MDT: Multi-disciplinary team
ADL: Activities of daily living
ASES: American shoulder and elbow surgeons
GCT: Giant cell tumors of bone
MGCT: Malignant giant cell tumors of bone
MSTS: Musculoskeletal tumor society scoring system
VAS: Visual analog scale
