# Supplementary material for: Use of LARS for soft tissue function reconstruction during tumor-type hemi-shoulder replacement achieves a good prognosis: a retrospective cohort study
Source: World J Surg Oncol. 2023 Apr 4;21:123. doi: 10.1186/s12957-023-03008-7 (PMC10071767; doi:10.1186/s12957-023-03008-7)
Supplement: Supplementary file 1 — Additional file 1: Figure S1. Postoperative follow-up revealed complications of prosthesis dislocation. (a) X-ray review indicated that the joint structure was stable at 6 months postoperatively. (b) Forward and upward dislocation of the prosthesis was found at 1 year postoperatively. (c) The red arrow indicates a dislocated humeral head in the patient's frontal view. [file 12957_2023_3008_MOESM1_ESM.docx]

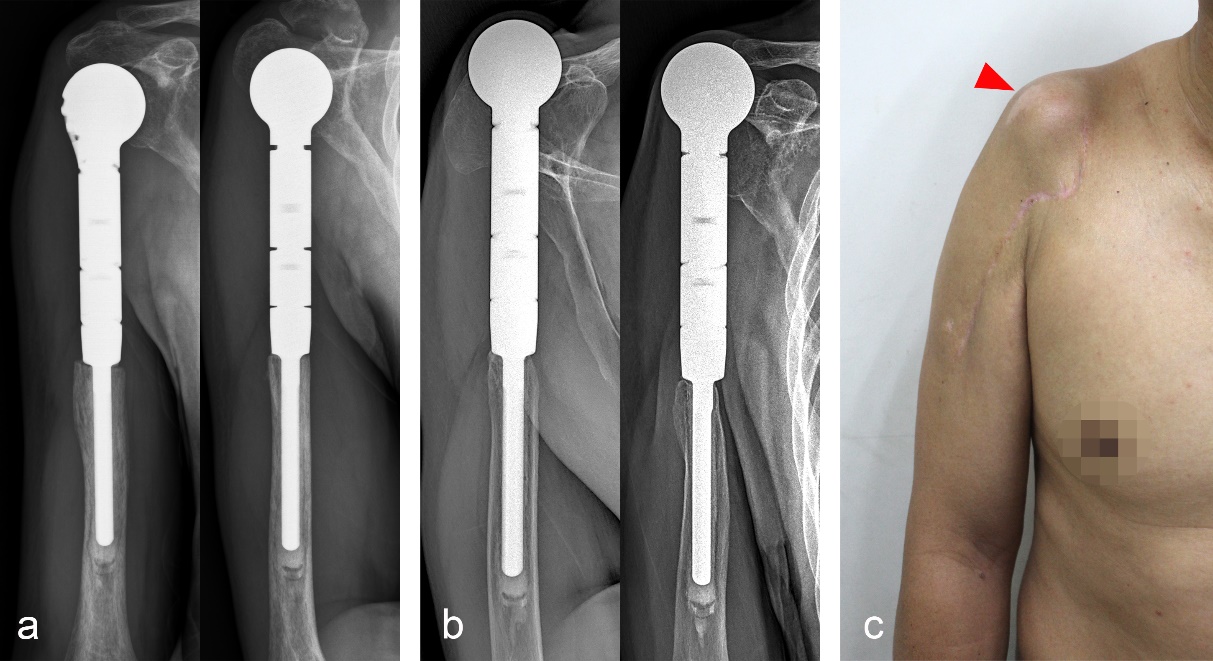


**Figure**. Postoperative follow-up revealed complications of prosthesis dislocation. (a) X-ray review indicated that the joint structure was stable at 6 months postoperatively. (b) Forward and upward dislocation of the prosthesis was found at 1 year postoperatively. (c) The red arrow indicates a dislocated humeral head in the patient's frontal view.
